# A novel experimental approach for nanostructure analysis: simultaneous small-angle X-ray and neutron scattering

**DOI:** 10.1107/S1600576720005208

**Published:** 2020-05-13

**Authors:** Ezzeldin Metwalli, Klaus Götz, Sebastian Lages, Christian Bär, Tobias Zech, Dennis M. Noll, Isabel Schuldes, Torben Schindler, Annemarie Prihoda, Herbert Lang, Jürgen Grasser, Mark Jacques, Luc Didier, Amrouni Cyril, Anne Martel, Lionel Porcar, Tobias Unruh

**Affiliations:** a Institute for Crystallography and Structural Physics, Friedrich-Alexander-Universität Erlangen–Nürnberg, Staudtstrasse 3, Erlangen, 91058, Germany; b Center for Nanoanalysis and Electron Microscopy (CENEM) and Interdisciplinary Center for Nanostructured Films (IZNF), Friedrich-Alexander-Universität Erlangen–Nürnberg (FAU), Cauerstrasse 3, Erlangen, 91058, Germany; c Institut Laue–Langevin, 71 Avenue des Martyrs, Grenoble, 38042, France

**Keywords:** small-angle X-ray scattering, small-angle neutron scattering, SAS, SANS, SAXS, nanomaterials

## Abstract

Exploiting small-angle X-ray and neutron scattering (SAXS/SANS) on the same sample volume at the same time provides complementary nanoscale structural information in two different contrast situations. Unlike an independent experimental approach, the truly combined SAXS/SANS experimental approach ensures the exactness of the probed samples, particularly for *in situ* studies. Here, an advanced portable SAXS system that is dimensionally suitable for installation in the D22 zone of ILL is introduced. The SAXS apparatus is based on a Rigaku switchable copper/molybdenum microfocus rotating-anode X-ray generator and a DECTRIS detector with a changeable sample-to-detector distance of up to 1.6 m in a vacuum chamber. A case study is presented to demonstrate the uniqueness of the newly established method. Temporal structural rearrangements of both the organic stabilizing agent and organically capped gold colloidal particles during gold nanoparticle growth are simultaneously probed, enabling the immediate acquisition of correlated structural information. The new nano-analytical method will open the way for real-time investigations of a wide range of innovative nanomaterials and will enable comprehensive *in situ* studies on biological systems. The potential development of a fully automated SAXS/SANS system with a common control environment and additional sample environments, permitting a continual and efficient operation of the system by ILL users, is also introduced.

## Abstract 2

A portable small-angle X-ray scattering instrument with geometrical dimensions suitable for installation at the D22 instrument was designed and constructed for simultaneous small-angle X-ray and neutron scattering experiments at ILL.

## 1. Introduction   

Small-angle scattering (SAS) of X-rays (SAXS) and neutrons (SANS) is a powerful experimental technique that is widely used in materials science, soft condensed matter and structural biology (Fitter *et al.*, 2006 ▸; Glatter & Kratky, 1982 ▸; Svergun *et al.*, 1987 ▸, 2013 ▸). The SAXS and SANS methods both provide statistically relevant structural information on length scales from 0.1 to 100 nm (Blanchet & Svergun, 2013 ▸; Koch *et al.*, 2001 ▸; Svergun, 2010 ▸). However, X-rays interact prevalently with the electron shell of the atoms, while neutrons interact with their nuclei, and thus the two radiations offer two different contrasts (X-ray and neutron scattering cross section). The SAS method has been used with great success in investigating the structure of soft condensed matter such as emulsions (Schmiele *et al.*, 2016 ▸), micelles (Schmutzler, Schindler, Goetz *et al.*, 2018 ▸; Schmutzler, Schindler, Schmiele *et al.*, 2018 ▸), liquid crystalline structures (Gehrer *et al.*, 2014 ▸; Schmiele, Gehrer *et al.*, 2014 ▸; Schönhals *et al.*, 2010 ▸) and organic nanoparticle (NP) dispersions (Schuldes *et al.*, 2019 ▸; Schmiele, Schindler *et al.*, 2014 ▸; Unruh, 2007 ▸). For advanced functional materials applications (*e.g.* sensors, solar cells and lithium-ion batteries), SAXS and SANS are important techniques to study the formation, growth and stabilization of inorganic nanomaterials (Schindler *et al.*, 2015 ▸, 2017 ▸; Wang *et al.*, 2015 ▸, 2019 ▸; Yin *et al.*, 2018 ▸; Zheng *et al.*, 2018 ▸; Mohl *et al.*, 2018 ▸; Futscher *et al.*, 2019 ▸; Metwalli *et al.*, 2015 ▸). In modern biological applications, the SAS method is an essential tool for the structural characterization of proteins, nucleic acids and lipids, as well as for monitoring structural changes during protein folding, intrinsic disorder, conformational transitions and protein–protein assembly processes (Fitter *et al.*, 2006 ▸; Svergun *et al.*, 2013 ▸; Blanchet & Svergun, 2013 ▸; Koch *et al.*, 2001 ▸; Schindler *et al.*, 2018 ▸). In contrast to analytical methods such as scanning or transmission electron microscopy (SEM/TEM), SAS offers a nondestructive bulk characterization technique allowing analyses of a small sample volume at very low concentrations (<10 mg ml^−1^) and a rapid data-acquisition capability (Svergun *et al.*, 2013 ▸). In other words, the dispersed materials can be easily and reliably characterized in solution using SAS without extensive preparation such as drying or plunge freezing (Frank, 2002 ▸), in contrast to other analytical methods where artifacts are likely to arise because of changes prior to characterization. By employing software tools that enable high-throughput data reduction and efficient data analysis, time-resolved SAS measurements can uniquely be utilized for studies on growth rates and phase transitions of colloidal dispersions in real-time mode (Graceffa *et al.*, 2013 ▸; Jensen *et al.*, 2010 ▸; Tanaka *et al.*, 2007 ▸; Arleth *et al.*, 2014 ▸; Illing & Unruh, 2004 ▸). Thus, SAS holds great promise for answering many unsolved scientific questions, especially for soft matter and structural biology research.

### 1.1. SAXS and SANS: contrast variations and wide-angle option   

Both SAXS and SANS provide information on global shape, size and size distribution as well as the spatial distribution of dispersed macromolecules in solution (Putnam *et al.*, 2007 ▸). In particular, they provide the diameter and mass of gyration of isotropically shaped particles, as well as the linear mass density and cross-sectional size of anisotropically shaped particles (Putnam *et al.*, 2007 ▸; Koch *et al.*, 2003 ▸). The internal structure of many biological systems and colloidal dispersions is complex, and such information is complementary to the global structural details (Whitten, Jeffries *et al.*, 2008 ▸; Grossmann *et al.*, 2008 ▸). While the SAXS method is sensitive to the electron-density difference between dispersed nano-sized moieties and their environment, the SANS method uses neutrons as probes which are sensitive to different isotopes of the same element, allowing variation of contrasts and internal structural determination. Thus, SAXS and SANS provide complementary information about the structure of the investigated samples. There are many aspects to the complementarity of SAXS and SANS (Schmidt, 1995 ▸; Zemb & Diat, 2010 ▸). One important aspect is contrast variation. For instance, contrast-variation SAXS has been used to investigate the internal electron-density structure of a system by employing chemical agents such as glycerol, sucrose, gadolinium-based molecules and salt (Grishaev *et al.*, 2012 ▸; Chen *et al.*, 2014 ▸; Kuwamoto *et al.*, 2004 ▸). But owing to the potential chemical and physical modifications of the samples upon incorporating these agents and their limited electron-density ranges, the utility of SAXS-based contrast-variation measurements is not substantial. Additionally, the viscosity of a liquid sample was reported to increase after using a high concentration of contrast agents such as sucrose (Chen *et al.*, 2014 ▸). In a viscous system, a potential transition from a non-equilibrated to an equilibrated state may affect the evolved nanoscale structure. Furthermore, the applicability of high salt concentrations for studying biological systems in contrast-variation SAXS is narrow because the salt may cause undesired chemical modifications (Chen *et al.*, 2014 ▸). For neutrons, the contrast-variation option is, however, more popular and can be achieved by targeted isotopic substitution of the nano­structured components or by changing the ratio of deuterated/protonated solvent of the investigated samples (Neylon, 2008 ▸; Sugiyama *et al.*, 2016 ▸). Using contrast-variation SANS, the resulting scattering profile of a particular component and its relative orientation in a multicomponent assembly can be retrieved (Whitten, Cai & Trewhella, 2008 ▸; Schmutzler, Schindler, Schmiele *et al.*, 2018 ▸). This would reveal molecular recognition and the unique relative positioning of the components within nanoscale assemblies (Svergun & Nierhaus, 2000 ▸). In other words, contrast-variation SANS is a uniquely suited method for probing the structure of a particular component in the presence of another, by employing a contrast-matching experimental approach. The wide-angle option of the neutron scattering method is limited in investigating atomic and small-sized structures due to the broad wavelength band (Δλ/λ ≃ 10%) that is typically employed for SANS instruments. At the National Institute of Standards and Technology, a high-resolution (Δλ/λ ≃ 1%) SANS instrument is available for investigating a large *q* range (molecular resolution) but at a highly reduced flux. Alternatively, X-ray scattering reliably enables a wide-angle scattering (WAXS) option, which satisfactorily provides information on the atomic resolution and finer structural features of the investigated systems.

### 1.2. Combined SAXS and SANS: independent measurements   

From the above discussion it is obvious that SAXS and SANS are truly complementary techniques that access a wide *q* range for structural information – from atomic and mol­ecular scales to large assemblies. Despite the SANS technique’s unique advantage in internal structural determination (Schindler *et al.*, 2015 ▸), many multicomponent entities in an assembly continue to pose a considerable challenge to neutrons. Thus, the SAXS technique is used to obtain comprehensive structural details. The need to combine SAXS and SANS to resolve structural information has been reported for many scientific cases (*e.g.* core–shell colloids, biological systems, nanoparticle–protein complexes) (Schindler *et al.*, 2015 ▸, 2018 ▸; Hennig *et al.*, 2013 ▸; Spinozzi *et al.*, 2017 ▸; Schuldes *et al.*, 2019 ▸). As an example, for the suspension of organic coated solid inorganic NPs (Schindler *et al.*, 2019 ▸, 2015 ▸, 2017 ▸), the SANS method is particularly sensitive to the hydrogenated organic shell while the core is transparent to neutrons. Thus, SANS will provide the form factor of the shell in a representative hollow-shell-like particle (Schindler *et al.*, 2019 ▸). In contrast, SAXS measurements of the same core–shell sample are sensitive to the high-electron-density core and significantly less sensitive to the shell. The structures of both shell and core can thus only be obtained via complementary SAXS and SANS methods. For organic core–shell systems, the opposite scenario is true. For systems such as surfactant (sodium dodecyl sulfate)-coated cetyltrimethylammonium bromide micelles, the shell is invisible to the SANS method owing to enrichment of employed D_2_O in the shell (Bergström & Pedersen, 2000 ▸). The form and structure factors of the hydrogenated core can thus be obtained. For this organic core–shell sample, the SAXS method, however, will provide structural information on the shell-like structure owing to the high-electron-density counter-ions in the electrical double layer within the shell. Thus, SAXS and SANS can together provide detailed structural information on many systems, especially for *in situ* studies. For biological systems, combined SAXS/SANS investigations have become an established tool to obtain a complete structural picture together with computer-based structure calculation protocols. For instance, contrast-variation SANS investigation of selectively deuterated subunit samples, such as protein–RNA complexes, can provide valuable additional information on these subunits (Hennig *et al.*, 2013 ▸). Ambiguities in the structural models obtained from analysis of the SANS data can be further resolved by a SAXS experiment, which should yield information about the overall shape of the complex molecular structure (Hennig *et al.*, 2013 ▸). The importance of joint SAXS/SANS experiments has already been recognized. For instance, an initiative (SAS platform) at two large-scale facilities (ILL and ESRF) has been launched, allowing joint beamtime allocation on both SAXS (BM29–ESRF) and SANS (D22–ILL) instruments (Lapinaite *et al.*, 2013 ▸).

### 1.3. Combined SAXS and SANS: simultaneous mode   

An analytical concept based on coupling of SAXS and SANS methods in a single experiment is an important step forward in the direction of novel analytical technologies that pave the way to innovations in advanced nanostructured materials. The key advantage of simultaneous SAXS/SANS measurements is the ability to probe a particular sample at the same time in two different contrast situations for X-rays and neutrons. It is almost impossible to check if two samples are really identical by performing independent SAXS and SANS measurements, especially at different contrast-variation levels (deuteration and buffers). However, simultaneous SAXS/SANS measurements both ensure the exactness of the probed samples and allow the potential of using simultaneous structural analysis models for both SAXS and SANS data. The combined SAXS and SANS technique established here not only provides the ability to probe the same sample volume at the same time but also allows access to temporal development of nanoscale-structured materials in two different contrast situations for X-rays and neutrons. This technique offers the unique advantage of accessing the cross-correlation of two components when two different simultaneous contrast situations are probed for multicomponent samples during *in situ* experiments. For instance, simultaneous X-ray and neutron study will permit us to analyze and understand the complicated correlated nanostructures of two types of nanoscale components simultaneously under the influence of an external stimulus at different points of time. Consequently, this allows a straightforward and unambiguous interpretation of the scattering data using a unified structural model. The following examples will give a flavor of applications that will benefit from the newly established nanoanalytical tool:

(i) For solar cell applications, during the synthesis/function­alization of semiconductor NPs, a detailed insight into the complex relationship between the dimension of organic capping moieties (SANS) and the resulting NPs’ structure (SAXS) can be achieved.

(ii) For sensor applications, the structures of thermo-responsive microgel cores (SANS) and the metal NPs (SAXS) assembled on their surface upon swelling/deswelling under an external stimulus can simultaneously be resolved.

(iii) For catalyst materials, cross-correlation between the structure of the adsorbed reactants/products (SANS) and the pore morphology/crystalline state of a nanoporous catalyst (SAXS) under predefined conditions can be studied.

In this work, we will provide a technical description of a custom-made portable SAXS system that is geometrically suitable for installation at a SANS instrument (D22) of ILL. The whole system’s components are uniquely mounted on a single metal chassis so it can be craned and quickly moved inside the D22 zone. The SAXS instrument can be installed in the D22 zone within one hour, allowing a fast start of simultaneous SAXS/SANS experiments. Furthermore, a lead shielding configuration efficiently stops high-energy gamma radiation so that a minimum background on the X-ray detector is achieved. The X-ray and neutron beams are orthogonal and are superimposed on the same sample positioned at 45° with respect to both beams, allowing simultaneous SAXS/SANS measurements. We will present the full specifications of the new setup and the first scientific experiment using the simultaneous SAXS/SANS method. Finally, we will describe potential developments, which include further minimization of background radiation on the X-ray detector and a new control software within the ILL software tools (*NOMAD*), to facilitate combination of the SAXS and SANS methods for ILL users.

## 2. General description   

**Figure 1:**
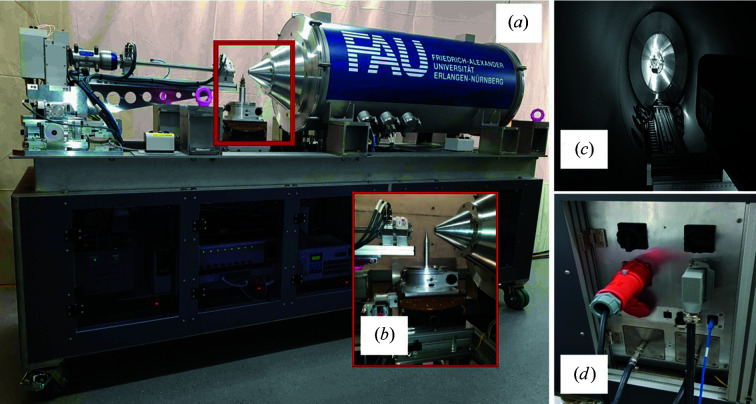
(*a*) Photograph of the portable SAXS instrument for simultaneous SAXS/SANS measurements at D22–ILL. (*b*) Six-axis goniometer for correct sample alignment with respect to both X-ray and neutron beams. (*c*) EIGER R 1M detector with three degrees of freedom to move inside the vacuum chamber. (*d*) The central hub for easy and smooth plug-and-play operation, including power supplies, cooling water and ethernet cable. All system components are enclosed within a single heavy-duty metal non-magnetic chassis.

An advanced SAXS system based on a Rigaku switchable copper/molybdenum microfocus rotating-anode X-ray generator and an EIGER R 1M detector with a changeable sample-to-detector distance (SDD) from 0.5 m up to 1.6 m in a vacuum chamber was designed and constructed (Fig. 1 ▸). The SAXS system is dimensionally suitable for installation at the D22 instrument of ILL.

With a Rigaku VariMax optics and a beam divergence control system directly connected to the X-ray source, switching from one wavelength to the other (Cu *K*α or Mo *K*α) is easily achievable. The evacuated collimation system of about 56 cm in length is composed of three fully automated vacuum slits. Each slit has four independently moveable blades which define the aperture size and position. One tungsten carbide and two scatterless slit systems equipped with Si and GaAs blades define a variable-size and low-divergence beam suitable for either Cu or Mo X-rays, respectively. The whole assembly (X-ray source, optics and collimation system) is firmly connected and can be moved along three axes (*x*, *y*, *z*) to enable the X-ray beam position to be fine-tuned such that both the X-ray and neutron beams can be superimposed at the sample position. A goniometer (Huber) with six degrees of freedom was employed to achieve highly flexible sample positioning. For the experiment presented here, the sample was positioned at 45° relative to both of the orthogonal X-ray and neutron beams.

**Figure 2:**
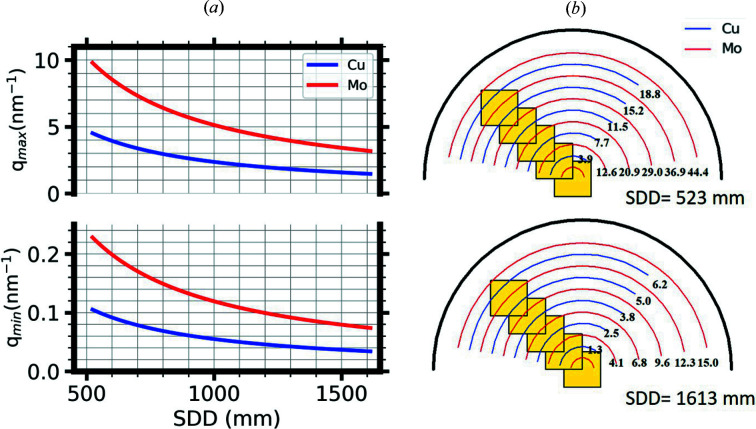
(*a*) Center and (*b*) off-center accessible *q* ranges at different SDDs for both Cu *K*α and Mo *K*α radiation. When the direct beam is on the detector’s active area (center position), *q*_min_ (the smallest scattering angle) is assumed to be at a value of 4 × FWHM of direct beam intensity. For the off-center configuration, scaled representations of the detector vacuum tube (black semicircle) and detector active area (yellow rectangles) for two different SDDs (523 and 1613 mm) are shown. The blue (Cu *K*α) and red (Mo *K*α) semicircles represent scattering rings at different accessible *q* values (nm^−1^) and their relative positions on the detector’s active area.

The scattering data are acquired by an EIGER R 1M detector located in an evacuated detector chamber. No beamstop is needed to stop the direct beam in front of the detector, enabling scattering data and direct-beam intensity measurements on the detector without any scattering from the beamstop. As a result, no correction to account for the beamstop shadow is required. All components of the system were mounted on a standalone metal rack (chassis) that made it easily movable for use on the D22 instrument. The D22 floor was equipped with an adjustable metal–concrete support base for the fast and precise positioning of the SAXS system in the D22 zone. The *Network Based Instrument Control System* (*NICOS*) software is employed for controlling all instrument motors and data acquisition. It is a Python-based user-friendly client–server run­ning on top of the *TANGO* environment (Kleines *et al.*, 2015 ▸). The detector allows the X-ray direct beam in either a center or an off-center position, permitting various *q* ranges (*q* = 4πsinθ/λ, where θ is half the scattering angle). When the beam is on the active area of the detector, the range of the scattering angle for Cu *K*α radiation of 8.048 keV (0.15406 nm) can be varied by selecting different SDDs, enabling an interactive *q* range between 0.040 and 4.4 nm^−1^ [Fig. 2 ▸(*a*)]. Mo *K*α radiation of 17.45 keV (0.0711 nm) can alternatively be used when a higher penetration depth of the radiation, or a larger *q* range (0.07–9.7 nm^−1^), is required [Fig. 2 ▸(*a*)]. Various larger *q* ranges can be further permitted by moving the detector to an off-center position in the vacuum chamber [see Fig. 2 ▸(*b*)].

## 3. SAXS configuration   

### 3.1. X-ray source and optics   

A dual-wavelength (DW) X-ray source that is based on the latest generation of the microfocus rotating-anode (Rigaku MicroMax-007 HFMR) generator was employed. By utilizing only one rotating anode, two switchable X-ray targets (Cu and Mo) are available in one compact system. The effective focus size is 70 µm, and the generator operates at 40 kV–30 mA for Cu (λ = 0.15406 nm) and at 50 kV–24 mA for Mo (λ = 0.0711 nm). In contrast to the Cu *K*α radiation typically used for soft matter samples, Mo *K*α is chosen for highly absorbing samples as well as further extended* q *range. To deliver monochromatic Cu *K*α or Mo *K*α beams, the X-ray source is coupled with auto-switching DW optics. Rigaku’s VariMax DW optics are composed of dual optics in a single compact housing. The optics employ two multilayer mirrors arranged ‘side by side’ (Montel optics) (Liu *et al.*, 2011 ▸).

### 3.2. Collimation system   

The collimation system, of length 56 cm, is composed of three compact slit systems (JJ X-ray). The first is the tungsten carbide (WC) slit system, which is suitable for both Cu *K*α and Mo *K*α radiation. It is placed directly after the optics. The other two motorized scatterless slit systems are integrated at the end of the fully evacuated collimation line. Owing to the short collimation length which has been constrained by the available space in the D22 zone, a two-slit collimation setup was finally employed, either W/Si or W/GaAs. The whole assembly (X-ray source, optics and collimation system) is placed on a three-axis table that enables a flexible adjustment of the X-ray beam position (*x*, *y*, *z*) to the same sample spot as probed by the neutron beam.

### 3.3. Sample cell   

The sample is located within an area of about 11 × 11 cm under ambient conditions. In the validation experiment, a six-axis goniometer was employed to adjust the sample position relative to both the X-ray and the neutron beams. A sample cell composed of two identical compartments was constructed. One compartment held the sample and the other was filled with silicon oil in which a PT100 temperature sensor was inserted to monitor the cell’s temperature. The sample cell system comprised a water-cooled copper holder and two attached copper blocks with an inner silver spacer that acted as a chemically resistant and thermally conductive enclosure for the sample. Mica windows (30 mm in diameter, 25 µm thick) were held in position by polyether ether ketone caps equipped with O-rings to ensure cell tightness. The windows formed a sample thickness of 1 mm in total. The sample was situated and aligned at 45° relative to both the X-ray and the neutron beams. A corrected optical path length of 1 mm × 2^1/2^ for both X-rays and neutrons is calculated.

### 3.4. Detector   

The windowless X-ray EIGER R 1M detector (Dectris) is composed of two modules arranged in horizontally oriented rows, yielding a total active area of 79.9 × 77.2 mm (array of 1030 × 1065 pixels, 75 µm pixel size). In the direction of the beam (*x* direction), the detector can move up to 1.6 m distance from the sample to allow extreme SAXS (*q*_min_ = 0.04 nm^−1^) or to 0.5 m distance allowing wide- or medium-angle X-ray scattering measurements (*q*_max_ = 4.4 nm^−1^). Furthermore, the transverse motion (*y*, *z*) of the detector enables the beam to be in either a center or an off-center position, permitting an extreme WAXS maximum *q* value of 40.0 nm^−1^ [Fig. 2 ▸(*b*)]. With a continuous readout with duty cycle >99% capabilities, the EIGER R is able to acquire short-duration exposures with a frame rate of 10 Hz. The transmitted beam intensity is continuously measured on the detector; no beamstop was used to stop the direct beam. With the EIGER’s auto-summation mode, the frames can be acquired at high frame rates on the pixel level, effectively avoiding any overflows. The detector is a single-energy threshold detector that suppresses low-energy X-rays (<4 keV).

### 3.5. Control software   

Device servers for both the EIGER R 1M (Dectris) detector and phyMOTION motor controllers were developed and implemented within the *TANGO* control system framework (Chaize *et al.*, 1999 ▸; Kleines *et al.*, 2015 ▸). *NICOS* was initially developed at FRM II by one of the co-authors and later maintained and developed by the Heinz Maier-Leibnitz Zentrum (MLZ) software group. It provides a user interface for performing scientific experiments, interacting with the underlying *TANGO* system. The *NICOS* user interface offers an online graphical view of the acquired data and interactive Python command scripting for fully automated experiments.

### 3.6. Portable SAXS at D22–ILL   

**Figure 3:**
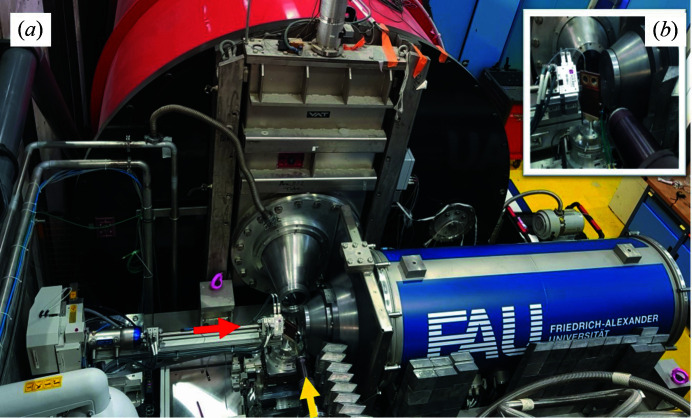
(*a*) Photograph of the SAXS instrument installed at the D22 instrument (ILL) for simultaneous SAXS/SANS experiment. (*b*) A sample at an angle of 45° relative to both orthogonal neutron and X-ray beams. Lead shielding can be seen (1) on the front of the detector vacuum chamber, (2) along the neutron collimation and (3) on the side of the vacuum chamber of the X-ray detector. The red and yellow arrows indicate the directions of the X-ray and neutron beams, respectively.

The transport flexibility of the current advanced laboratory-scale SAXS system opens up new opportunities for combining multiple complex and bulky analytical methods in a single experiment (Fig. 3 ▸). Targeted to be installed at the D22 instrument (ILL) for simultaneous SAXS/SANS measurements, the SAXS system consists of a portable heavy-duty chassis that hosts all components (source, detector generator, chillers, vacuum pumps, motor controllers, main remote servers, and electrical and mechanical units). The mobility aspect of this system makes it ideal not only as a system that is transportable to a remote location (D22–ILL) for *in situ* studies but also as a standby 24/7 complementary research tool at ILL, enabling independent SAXS experiments following neutron measurements for samples that are highly susceptible to fast aging.

Additionally, to ensure a quick initial adjustment of the system after the transport process inside the D22 zone, the floor of the D22 zone was equipped with two steel–concrete bases. The supporting base comprises a concrete-wrapped steel base plate and a detachable vertical steel plate perpendicular to the upper surface of the base plate. With the help of adjustment screws on these perpendicular plates, the SAXS chassis can be satisfactorily adjusted at 2.5 cm range distances. Safety interlocks and zone warning lamps for operating the new SAXS system in the D22 zone were already installed. Overall, the SAXS system is mobile in design and can be used as a plug-and-play instrument. Within one hour, the system can be craned; placed inside the D22 zone; connected to power supplies, cooling water and the internet; and then turned on for the alignment step. A conceptual difference of this current custom-made SAXS system with respect to commercial systems is its high flexibility in design, with all its main components (source, optics, collimation, sample and detector) freely moveable in all directions (*x*, *y*, *z)*. This flexibility is essential to probe the same sample volume using both X-ray and neutron radiation – placing the X-ray beam within ±1 mm of the same sample area where neutrons are impinging on the sample.

## 4. Performance evaluation of the portable SAXS system   

The bright DW (Cu and Mo) rotating-anode X-ray generator (Rigaku MicroMax-007 HFMR) and focusing optics (Rigaku’s VariMax DW) followed by a three-slit collimation system provide a photon flux of ∼1.1 × 10^7^ photons s^−1^ (for a 0.5 × 0.5 mm beam on the sample position). The new generation of the Rigaku source runs at a maximum power of 1.2 kW and rotation speed of 9000 r min^−1^ and has a small effective focal point of 0.07 mm. These characteristics facilitate a performance that approaches that of second-generation synchrotron bending-magnet beamlines. Because the SAXS system is targeted to be mounted/demounted upon beamtime allocations at the D22 instrument, important characteristics such as flux, space, mobility and quick start-up are prioritized. For instance, the beam brilliance of the currently employed microfocus rotating-anode generator source is six times greater than that of the state-of-the-art microfocus sealed tube (Skarzynski, 2013 ▸). Although the high beam brilliance of metal-jet-based sources developed by Excillum exceeds the brilliance of the current microfocus rotating-anode source, the lack of transport flexibility hinders the usage of these sources. The currently employed X-ray source provides the high flux and mechanical robustness needed for the current portable laboratory-scale SAXS system.

Additionally, the intensities of the X-ray and neutron beams are comparable and in synergy regarding possible measuring time. On the detector side, data quality, costs and the time needed for the data collection are major determining factors for the selected detector technology. With no dark current or readout noise, short readout time (10 µs), high spatial resolution (75 × 75 µm), and a relatively large sized active area (1030 × 1065 pixels), this detector is well suited for the intended combined SAXS/SANS experiments. Compared with the extremely fast readout (200 Hz) of Pilatus (Dectris) detectors suited for synchrotron radiation, the windowless and vacuum-compatible EIGER R detector with a frame rate of 10 Hz (100 µs frame time) is better suited for SAXS data collection comparable to the fast SANS measurements at D22. The whole system of optics, collimation and detector is evacuated to minimize air-scattering effects. However, at the sample position, a free space (air) is necessary to allow a spacious area for a perpendicular neutron beam (with respect to the X-ray beam).

**Figure 4:**
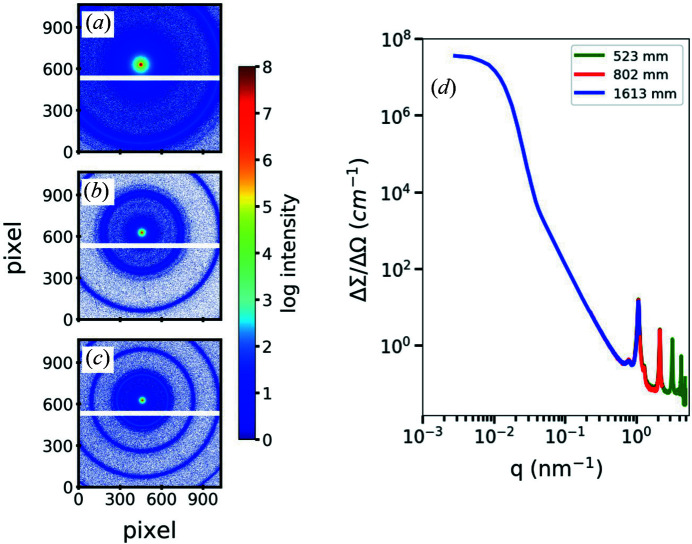
2D SAXS patterns of silver behenate (Agbh) at three different SDDs of (*a*) 1613 mm, (*b*) 802 mm and (*c*) 523 mm, indicating a SAXS *q* range between 0.04 and 4.4 nm^−1^. (*d*) Azimuthally averaged SAXS data of Agbh showing overlapping data along the three employed SDDs.

**Figure 5:**
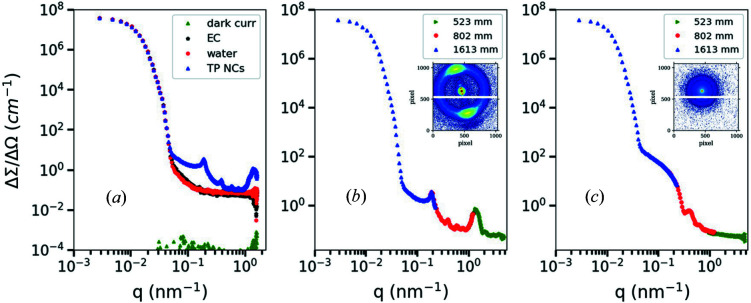
(*a*) Azimuthally averaged SAXS data of the empty cell (EC, black), a 1 mm-thick water layer (red) sandwiched between two mica windows (cell), the detector dark noise (green) and a sample of platelet-shaped tripalmitin nanocrystals (TP NCs) in water (blue) collected at SDD = 1613 mm. The 1D SAXS profiles of (*b*) platelet-shaped tripalmitin nanocrystals and (*c*) colloidal silica NPs (Ludox TM50; average size = 26 ± 2 nm) collected at different SDDs. Data were plotted and overlapped over different *q* ranges on an absolute scale. The insets are the corresponding 2D SAXS patterns.

The performance of the portable SAXS instrument was examined using calibrants and reference samples. For instance, glassy carbon, as a secondary intensity standard, and silver behenate [CH_3_(CH_2_)_20_COOAg] have been used to calibrate the absolute intensity scale and the *q* scale, respectively. The 2D SAXS scattering patterns of silver behenate at three different SDDs are displayed in Fig. 4 ▸. With the beam in the center position on the detector, the scattering pattern with different *q* ranges can be acquired for SDDs between 0.5 and 1.6 m. Owing to perfect *q* calibration, peaks accurately overlap for all the measurements [Fig. 4 ▸(*d*)]. Two aqueous suspensions of organic and inorganic nanoparticles were used for testing our system. The first sample is an aqueous suspension of platelet-shaped tripalmitin nanocrystals (TP NCs). Corresponding systems of TP NCs have been intensively studied by our group (Schmiele *et al.*, 2015 ▸; Schmiele, Gehrer* et al.*, 2014 ▸) as potential drug delivery systems. The second sample is an aqueous dispersion of commercial silica particles (Ludox TM50, 50 wt%) purchased from Sigma–Aldrich; the average diameter of the SiO_2_ NPs is about 26 ± 2 nm (see fitting results in Fig. 1S of the supporting information). These samples were selected because they provide two different X-ray scattering powers (organic and inorganic particles) and cover essentially different *q* ranges (Fig. 5 ▸).

The dispersions were filled into a sample cell having a 1 mm-thickness gap sealed on both sides by two thin parallel mica windows. The sample holder was installed at the SAXS system in an air gap of about 11 cm. It was also apparent that the corrections for background and detector noise have minimal effect on the data. With a beam size of 0.5 × 0.5 mm on the sample position and a divergence of about 0.4 mrad at the detector position (SDD = 1612 mm), the apparent minimum value of *q* for which usable intensity can be obtained is 0.04 nm^−1^. Such a low *q*_min_ despite the short collimation is achievable owing to the absence of a beamstop. When the beam is in the center position on the active area of the detector, two SDD distances give access to a *q* range between 0.04 and 4.4 nm^−1^ [Fig. 2 ▸(*a*)], while the 2θ values are in the range between 0.2 and 6.5° (Fig. 2S). An off-center detector configuration (direct beam is not on the active area of the detector) can further be used for a wide-angle scattering experiment, permitting a maximum *q* of about 18.5 and 40.0 nm^−1^ [Fig. 2 ▸(*b*)] for Cu *K*α and Mo *K*α radiation, respectively.

## 5. SAXS/SANS setup   

The SAXS system has fulfilled all requirements including geometrical constraints, transport flexibility and safety aspects for installation at the D22 instrument. However, to ensure feasible and reliable operation, further challenges need to be overcome: (1) managing high-energy gamma and X-ray radiation levels at the ILL instrumental zones to acquire low-background 2D scattering patterns on the X-ray detector, (2) validating the SAXS and SANS data for the samples collected at a 45° angle with respect to those collected at 90° (typical sample angle for conventional scattering experiments), and (3) enabling a unified control of both SAXS and SANS experiments using user-friendly ILL software tools as well as developing an appropriate SAXS/SANS data reduction/analysis software using unified data set formats and fitting models. In the current work, we emphasize only the technical aspects of the SAXS system and the realization of combined SAXS/SANS measurements in one experiment. The software development of the state-of-the-art simultaneous SAXS/SANS method is in progress and not the scope of the current work.

### 5.1. SAXS and background radiation at D22   

**Figure 6:**
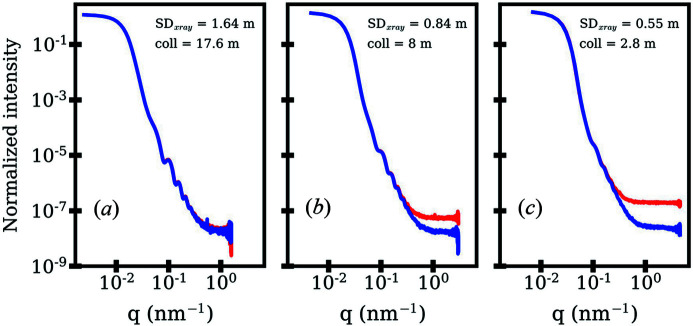
1D SAXS profiles calculated from the 2D detector images of silica NPs (100 nm) recorded with an open (red; simultaneous SAXS/SANS mode) and a closed (blue; standalone SAXS mode) neutron shutter for different neutron collimation lengths and X-ray sample-to-detector (SD_xray_) distances. Owing to the reduced neutron flux, the radiation background is insignificant on the X-ray detector for (*a*) a long collimation length, while for (*b*) intermediate and (*c*) short collimation lengths significant background radiation is observed for *q* > 0.31 nm^−1^. The intensity is plotted as an intensity normalized to the direct beam intensity with no background radiation subtraction for comparison purposes.

We explored the spatial distribution of high-energy gamma radiation in the D22 zone and constructed a preliminary experimental lead shielding around the SAXS system. This includes radiation protection walls with a thickness of 7–10 cm at the zone walls (between the D22 zone and other experimental zones), as well as lead shielding along the neutron collimation (Fig. 3 ▸). On the front side of the evacuated detector chamber of the SAXS setup, we designed, constructed and installed a cone of lead-based (PbSb_4_) material. In addition, a lead wall was installed on the SAXS chassis facing the neutron guide (Fig. 3 ▸). Extensive preliminary test experiments on the SAXS detector at D22, with the neutron source shutter either open or closed, were performed at different neutron collimation lengths and X-ray SDDs. Following the installation of the lead shielding, a significant reduction of the background radiation on the SAXS detector was achieved. In particular, a radiation background level competitive with the SAXS setup outside a neutron facility could be achieved for long neutron collimation lengths. Fig. 6 ▸ compares the 1D SAXS scattering profiles of monodisperse 100 nm silica NPs for both open (red) and closed (blue) neutron shutters at different neutron collimation lengths and X-ray SDDs. The highest background radiation has been observed for a configuration with a short neutron collimation length (2.8 m) and a small X-ray SDD (0.5 m) [Fig. 6 ▸(*c*)].

The current results indicate, however, the need for further reduction in background radiation (about 3–10 times) for intermediate and short neutron collimation lengths. Very recently, a double-threshold energy detector has been developed by Dectris (EIGER2 X 1M), and this offers an optional upgrade for further improvements (see Section 7). The preliminary results on this detector (data are not shown) operated in the D22 zone have confirmed that an efficient background-radiation reduction of about 10–15 times by employing, for instance, an energy window (7–9 keV) is better suited for the employed option of the Rigaku X-ray source with 8 keV X-rays. As part of the development process, such a detector will be purchased and installed. If this detector is employed together with further improvements related to the optimum shielding configurations, state-of-the-art laboratory SAXS data quality for all currently employed SANS setups at D22 for all operation conditions (collimation length, aperture and various beam fluxes) should be achievable without a special background subtraction.

### 5.2. SAS at two different sample angles   

**Figure 7:**
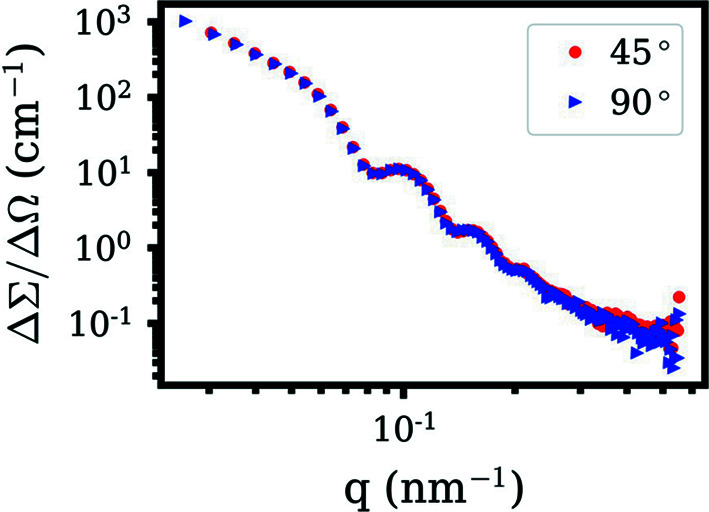
1D SANS profiles of 100 nm silica NPs collected at two different sample angles. The scattering profile corrected by multiplying each data point of the sample measured at a 45° angle by 1 mm × 2^1/2^ is perfectly coincident with that collected at a 90° angle.

For the SAS method, the beam typically falls on the sample at a normal incidence. For the combined SAXS/SANS experiment, both X-ray and neutron beams need to simultaneously illuminate the same sample volume. Thus, the sample should be positioned at an angle that allows dual-beam-based SAS investigations. One possible simple geometry is to place a cuvette at a 45° angle relative to both orthogonal X-ray and neutron beams. Our results indicate that a simple geometrical correction (sample thickness × 2^1/2^) can be used to replicate the X-ray (Fig. 2S) and neutron (Fig. 7 ▸) scattering data of a conventional normal-incidence-beam experiment.

### 5.3. *In situ* simultaneous SAXS/SANS experiment   

**Figure 8:**
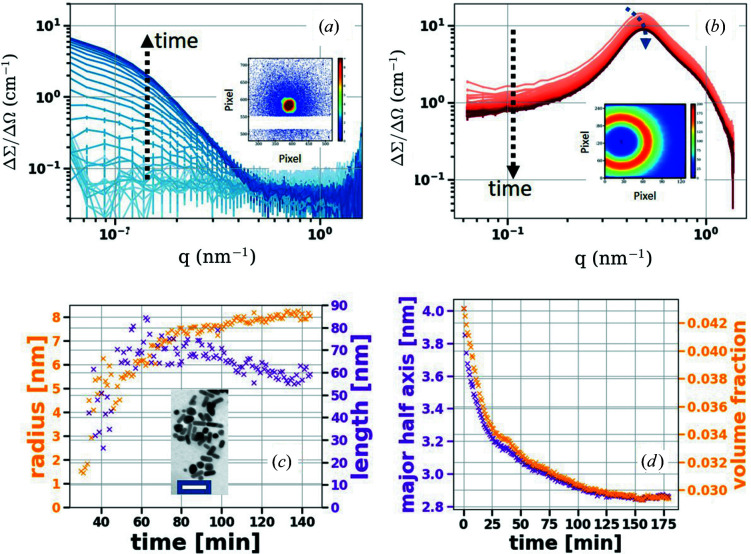
Time evolution of (*a*) SAXS and (*b*) SANS 1D profiles during the *in situ* reduction process of a gold solution at 308 K. Insets show 2D (*a*) SAXS and (*b*) SANS patterns during hydroquinone synthesis of Au NPs. (*c*) Rod-like Au particle radius and length as a function of reaction time, as revealed from the SAXS data fitting. The inset in (*c*) is a TEM image of the Au particles, with average radius and length of about 8 and 60 nm, respectively; the scale bar is 100 nm. (*d*) The exponential volume fraction decay of the CTAB micelles with time, as obtained from SANS data fitting.

The first experiment using the simultaneous SAXS/SANS technique has been successfully performed at D22–ILL. The cationic micelle structure development and evolved Au NP growth behavior during Au NP synthesis, via a seed-mediated growth procedure, were simultaneously monitored to emphasize the capabilities of the truly combined SAXS and SANS methods. Related protocols for preparation of rod-shaped Au NPs have previously been published by our group (Schmutzler, Schindler, Schmiele *et al.*, 2018 ▸; Schmutzler *et al.*, 2019 ▸). The Au NPs were synthesized using a gold precursor (HAuCl_4_) that forms small seeds after an initial reduction step using the reducing agent NaBH_4_ in the presence of cetyltrimethylammonium bromide (CTAB). Subsequently, the reduction of HAuCl_4_ using hydroquinone [C_6_H_6_(OH)_2_] in the presence of AgNO_3_ was performed using CTAB. The seed particles resulted in the formation of single-crystalline rod-shaped Au NPs. Within 2 h, stabilized large Au NPs were obtained, mostly during the reduction step, at a marginally elevated temperature (∼35°). The structure of the prepared sample was examined using simultaneous SAXS/SANS methods probing the same sample volume. This should provide new insights into possible structural reorganization of the stabilizing CTAB agent concomitant with the gold particle growth. Using the simultaneous SAXS/SANS method, the evolved particle growth (SAXS) and the CTAB structural modifications (SANS) were monitored at a time resolution of 1 min. The X-ray scattering power of gold (SLD = 1.24 × 10^−4^ A^−2^) is much higher than that of CTAB micelles (SLD = 9.2 × 10^−6^ A^−2^). Therefore, the gold NPs’ X-ray scattering is dominant in SAXS. For SANS, the CTAB micelles (−2.4 × 10^−7^ A^−2^) in deutrated water (6.38 × 10^−6^ A^−2^) are highly contrasted. The time evolution of the 2D SAXS and SANS patterns is visualized in the insets of Figs. 8 ▸(*a*) and 8 ▸(*b*). The characteristic SAXS signal of Au NPs becomes distinguishable at about 55 min into the synthesis process [Fig. 8 ▸(*a*)]. As revealed by the SANS data, the characteristic structural peak of the CTAB micelles [Fig. 8 ▸(*b*)] at *q* = 0.46 nm^−1^ was slightly shifted to the higher value of 0.50 nm^−1^ in a few minutes. Subsequently, the initial fast decay of the SANS signal was significantly decelerated when the characteristic gold SAXS signal was first detected after about 55–60 min. The SANS signal then diminished only slightly and leveled out at around 120 min. Interestingly, this coincides with a cessation in the SAXS intensity change (at 120 min). The SANS and SAXS results indicated a significantly reduced volume fraction of micelles [Fig. 8 ▸(*b*)], concomitant with progressive development of the Au NP shape and size within the first 55–60 min [Fig. 8 ▸(*a*)]. All details of sample preparation, the employed model and the data-fitting procedure are described in the supporting information. The SANS signal reached 1/*e* of its initial intensity after about 55 min, as it did not show any significant structural modifications during particle formation. In contrast, the SAXS signal intensity continued to develop up to 120 min into the synthesis. The Au particle structure extracted from the SAXS data can be described as mostly rod-shaped or elongated particles with an average width and length of about 8 and 60 nm, respectively.

A prominent Au particle size development can be particularly assumed during the first 55–60 min. The latter stages of Au NP growth above 60 min can be interpreted as a spontaneous restructuring phase of the evolved gold nanoparticles, where gold atoms and seeds are rapidly colliding together, forming large particles – as indicated by the enhanced SAXS signal intensity after 55 min. For the Au NPs, the initial growth within the first 55 min could not be detected owing to the low scattering volume. It seems that the incorporation of CTAB micelles in a structurally different stabilization shell is happening in the early stages of the synthesis. During the formation of large Au NPs, the CTAB micelle characteristic SANS signal dramatically diminishes. Then it is slightly changed, while the volume fraction of structurally well defined Au particles continuously increases (starting at 55–60 min) via a collision mechanism (Schmutzler *et al.*, 2019 ▸).

Therefore, it is apparent that potential structural reorganization of the stabilizing agents during the NP formation is not a major player in determining the size/shape of the evolving Au NPs. Our previous studies have already confirmed that the micelle structure influences the size and aspect ratio of gold NPs and can influence the stabilization of Au NPs in solution, depending to a large extent on their morphology (Schmutzler *et al.*, 2019 ▸). Consistently, the observations made in this measurement mean that, even though the Au NPs grew drastically (55–60 min), the micellar structure, residing at the surface of Au NPs or free in solution, is maintained. This clearly rules out particle stabilization mechanisms solely via formation of bilayers on the particle surface, which has been discussed in the literature for a long time. Indeed, the stability of the intermediate Au NPs is established via an attachment of the CTAB micelles themselves from solution to the surface of the Au particles. The so-attached micelles might regulate the Au NP growth relative to certain crystal facets via steric stabilization and manipulate the reaction and the subsequent attachment of gold ions to the surface, therefore driving an anisotropic metal growth.

So far, many inconsistent and controversial structural models from studies on the correlation between the size/shape of the structures formed by the organic stabilizer (*e.g.* CTAB) and the evolved inorganic NPs’ (*e.g.* Au) morphology have been reported (Karayil *et al.*, 2015 ▸; Ye *et al.*, 2012 ▸). This is due in part to the poorly studied interplay between the organic stabilizer and Au NPs during the synthesis step. The current results support our recent theory regarding the role of the micelle structure on the evolved particle morphology.

## 6. Conclusions   

Combining SAXS and SANS methods not only prompts a synergy that results when data sets are obtained simultaneously from the same sample volume but also allows access to the cross-correlation of phases/components when two different simultaneous contrast situations are probed for multiphase/component samples, ideally during *in situ*/real-time experiments. Moreover, the SAXS/SANS system will enable contrast-dependent successive-length-scale characterization of novel nanomaterials, addressing unsolved scientific questions. This study has presented the technical characteristics of a newly established portable SAXS apparatus that is dimensionally suitable for implementation at the D22 instrument of ILL, enabling combined SAXS and SANS measurements. The SAXS instrument is based on a high-flux rotating-anode generator with selectable Mo/Cu *K*α radiation, enabling nanoscale structural studies of soft materials and biological systems. The SAXS system comprises a collimation system including Montel multilayer optics and scatterless slits, a sample stage with six motional degrees of freedom, an evacuated beam path combined with an ambient sample holder, and an EIGER R 1M detector. The source–sample–detector geometry resulted in scalability and flexibility to expedite the alignment step of the X-ray beam on a distinct sample volume which is probed by neutrons. A heavy-duty non-magnetic metal chassis was employed to incorporate all the system’s components. The SAXS apparatus is mobile and peripheral in design to support plug-and-play operations, enabling its quick (<1 h) installation at the D22 instrument. Software control based on the *NICOS* user interface on top of a *TANGO* interface is used to control all motors and collect data in a fully automated manner. The assessment of the SAXS performance has been presented here for Cu *K*α radiation. Alternatively, Mo *K*α radiation can be used as needed for alternative sample geometries. A wide-*q* dynamic range can be achieved via a movable detector inside the vacuum chamber. Background intensity on the SAXS detector mainly caused by hard gamma rays can be efficiently minimized by effective optimized lead shielding. This study has presented a test experiment to emphasize the unique capability of *in situ* SAXS/SANS studies for determination of structural rearrangements during materials processing such as controlled nanoparticle growth. Exemplarily, the structural determination of both the hard (Au NPs) and soft moieties (CTAB) of surfactant-stabilized Au particles during the Au NP growth process was performed. The temporal evolution of the particles’ shape and size, their stabilization layer, and structural rearrangements of the CTAB micelles can only be determined and temporally correlated by using simultaneous SAXS and SANS methods.

## 7. Future developments   

Optimization for fully automated and simple-to-operate simultaneous SAXS/SANS measurements is a requirement to allow routine operation at D22 (ILL). The ultimate aim is to offer a simultaneous SAXS/SANS method as a widely used standard method for soft matter research and beyond at ILL. Thus, further developments, including sample environments, multi-sample changers, optimized routine operation and user-friendly software, should be considered in future research. Some of the essential sample environments (Jordan *et al.*, 2016 ▸; Lopez *et al.*, 2018 ▸; Wang *et al.*, 2004 ▸; Weigandt *et al.*, 2011 ▸) that are currently employed at ILL could be modified/developed to fit our system and to satisfy the demand of the ILL user community. However, a multiple-sample changer with temperature control should be constructed for routine sample measurements. Furthermore, it is proposed that sample environments such as high-performance liquid chromatography and flow cells that are already available at ILL should be modified to fit the combined setup to simultaneously collect X-ray and neutron data on biological samples, such as protein samples prone to aggregation. A detector with a double energy threshold will be purchased, enabling both low- and high-energy thresholds to suppress the background radiation on the X-ray detector near the employed X-ray energy. Moreover, standard data acquisition/monitoring routines, data formats and relevant data analysis software have to be developed. The optimization process also includes the standardization of the SAXS operation for flexible monitoring/data acquisition within the ILL software tools. In particular, a common flexible control software interface with several predefined standard configurations to enable small-, medium- or wide-angle X-ray scattering operation synchronized with the SANS data acquisition needs to be developed.

## 8. Related literature   

The following additional literature is cited in the supporting information: Breßler *et al.* (2015 ▸); Doucet *et al.* (2019 ▸); Hayter & Penfold (1981 ▸); Vigderman & Zubarev (2013 ▸); Zhang *et al.* (2010 ▸).

## Supplementary Material

Supporting information file. DOI: 10.1107/S1600576720005208/po5160sup1.pdf
