# Supplementary material for: A novel experimental approach for nanostructure analysis: simultaneous small-angle X-ray and neutron scattering
Source: J Appl Crystallogr. 2020 May 13;53(Pt 3):722–33. doi: 10.1107/S1600576720005208 (PMC7312133; doi:10.1107/S1600576720005208)
Supplement: Supplementary file 1 [file j-53-00722-sup1.pdf]

A Novel Experimental Approach for Nanostructure Analysis: Simultaneous Small Angle X-ray and Neutron Scattering (SAXS/SANS) Authors

Ezzeldin Metwalli[a], Klaus Götz[ab], Sebastian Lages[a1], Christian Bär[a], Tobias Zech[ab], Dennis M. Noll[a], Isabel Schuldes[a], Torben Schindler[a], Annemarie Prihoda[ab], Herbert Lang[a], Jürgen Grasser[a], Mark Jacques[c], Luc Didier[c], Amrouni Cyril[c], Anne Martel[c], Lionel Porcar[c] and Tobias Unruh[ab]*

[a]Institute for Crystallography and Structural Physics, Friedrich-Alexander-Universität Erlangen-Nürnberg, Staudtstr. 3, 91058, Germany

[b]Center for Nanoanalysis and Electron Microscopy (CENEM) and Interdisciplinary Center for Nanostructured Films (IZNF), Friedrich-Alexander-Universität Erlangen-Nürnberg (FAU), Cauerstr. 3, Erlangen, 91058, Germany

[c] Institut Laue-Langevin, 71, Avenue des Martyrs, Grenoble, 38042, France

Correspondence email: tobias.unruh@fau.de

[1]Current address: Department of Physics, Chalmers University of Technology, SE-412 96 Göteborg, Sweden

IMPORTANT: this document contains embedded data - to preserve data integrity, please ensure where possible that the IUCr Word tools (available from http://journals.iucr.org/services/docxtemplate/) are installed when editing this document.

1. Experimental

1.1. Materials:

Hydrogen tetrachloraurate trihydrate ($HAuCl_4 \cdot 3H_2O$, 99.99%, Alfa Aesar), hexadecyltrimethylammonium bromide (CTAB, for molecular biology, ≥ 99%, Sigma-Aldrich), silver nitrate ($AgNO_3$, ACS, 99.9 %, Alfa Aesar), n-hexanol (≥ 99%, Sigma-Aldrich), hydroquinone (HQ, ≥ 99%, Sigma-Aldrich) and sodium borohydride ($NaBH_4$, ≥ 98%, Merck KGaA) were used without further purification.

1.2. Particle Synthesis:

All solutions were prepared in deuterium oxide (D_2O, ≥ 99.9%, Eurisotop). To synthesize the initial seed particles, a 7.5 mL aqueous, 0.1 mol/L CTAB, and 0.1 mol/L hexanol solutions were prepared and kept at 35 °C. A volume of 0.2 mL of 0.010 mol/L $HAuCl_4$ was added, which resulted a light orange solution which stems from the complexation of gold with bromide ions. Under vigorous stirring 0.6 mL of freshly prepared 0.010 mol/L $NaBH_4$ was injected changing the liquid into a brown color. The solution was left undisturbed for 30 minutes to give enough time for the $NaBH_4$ to completely decompose. Due to presence of hexanol in the seed solution, the aggregation of gold seed particles can be slowed down immensely. This way the seed solution can be used for several weeks as has previously been demonstrated (Schmutzler *et al.*, 2019). This way, the resulting Au NPs solutions can be compared with one another without any noticeable aggregation of the seed particles, enabling a much larger timeframe in which the experiments can be carried out. The in-situ experiments were performed in the custom-made SAXS/SANS sample holder with two compartments and thermally controlled copper block. Volumes of 25 µL of 0.1 mol/L $HAuCl_4$ and 25 µL of 0.1 mol/L $AgNO_3$ were added to a 7.2 mL solution containing 0.1 mol/L CTAB turning it orange. Hydroquinone was chosen as a reducing agent, because of the slower reaction kinetics as has been shown in a previous study (Vigderman & Zubarev, 2013). Following this step, a 250 µL of 0.1 mol/L HQ solution was added to the mixed solution and shacked until it turned colorless. Following the addition of 1 mL of seed solution, the slow growth of AuNP begins. The SAXS sample holder was quickly filled via syringe through one of the three 0.5 mm holes from the top of the sample holder so that the sample was sandwiched between the mica windows. The other 0.5 mm holes serve to avoid bubble formation during sample loading. Following sample injection, the three holes were properly sealed using a thin film of pressure-sensitive adhesive to minimize solution evaporation and keep the sample concentration unchanged during experiments. The sample was situated and aligned at 45° relative to both the X-ray and the neutron beam. The temperature during the in-situ SAXS/SANS measurement was set at 35°C. The sample was measured for 3h. The particle solution was removed from the sample cell via a syringe and centrifuged at 9000 rpm for 30 minutes. The supernatant was removed and the sedimented nanoparticles (NPs) re-dispersed in D_2O for further ex-situ TEM analysis.

1.3. Simultaneous SAXS/SANS setup:

SANS measurements were performed on beamline D22 at the ILL (Institut Laue–Langevin, Grenoble, France). Wavelength at λ = 0.6 nm, a collimation of 7 m and a sample-to-detector distance (SDD) of 8 m were used for the in-situ study. An acquisition time of 60 s was used at these conditions in synchronization with the SAXS measurements. The SAXS images were simultaneously acquired using the installed SAXS system at the D22 zone. By employing Cu $K\alpha$ source with a photon flux of ≈ 1.1× 10^7 photons/s. For in situ simultaneous measurements, the sample-to-detector distance (SDD) was 1643 mm. All measurements were performed at 35 °C. The data have been corrected for sample thickness, acquisition time, and transmission. For 100-nm silica NPs, SANS measurements were performed at SSDs of 2.8, 8 and 17.6 m together with collimations of 2.5, 7 and 17.6 m, respectively.

1.4. SAXS and SANS data Analysis:

All SAXS measurements were for transmission, time and thickness corrected and absolute calibrated to 1 mm glassy carbon slab that was characterized at APL (Zhang *et al.*, 2010). The investigated nanostructures are assumed to exhibit a well-defined size and shape and have a homogeneous scattering length density. The scattered intensity displays the absolute scattering cross section $d\Sigma/d\Omega$ of a dispersion of NPs with interparticle interactions, which is described as (Schmutzler *et al.*, 2019):

$$\frac{d\Sigma}{d\Omega}(Q) = \frac{1}{V} < |F(Q)|^2 > S(Q) \ ,$$

where $F(Q)$ is described as the form factor, $S(Q)$ is the structure factor and Q the absolute of the scattering vector $\vec{Q} = |\vec{k_\iota} - \vec{k_s}|$. For elastic scattering the relation can be rewritten as $Q = 4\pi/\lambda\ sin(\Theta)$. The previous parameters are corresponding to the incident wave vector $\vec{k_\iota}$, scattered wave vector $\vec{k_s}$, wavelength of the probe particle λ and scattering angle Θ.

From TEM images could be seen that there was a mixture of gold nanorods and strongly anisotropic particles inside the final solution. The scattering signal could be modeled best with a cylinder form factor by fitting length and radius. When neglecting interparticle interactions, the form factor is described as follows:

$$\frac{d\Sigma}{d\Omega}(Q) = \frac{volfrac}{V} \int_0^{\frac{\pi}{2}} F^2(R, L, Q, \alpha) \sin(\alpha)\, d\alpha + B \quad ,$$

$$F(R, L, Q, \alpha) = 2(\Delta\rho)V \frac{sin\left(\frac{1}{2}QL\ cos(\alpha)\right)}{\frac{1}{2}QL\ cos(\alpha)} \frac{J_1(QRsin(\alpha))}{QRsin(\alpha)}$$

whereas R is the radius and L the length of the cylinder, α describes the orientation of the cylinder to the Q-vector and orientationally averaged by the integral, V is the volume of the cylinder $\pi R^2 L$, $\Delta\rho$ the scattering contrast between dispersion medium and particle, J_1 is the first order Bessel function,

$volfrac$ is the volume fraction of particles in the observed volume and B a constant describing the residual background. Furthermore, radius R and length L where distributed according to a gaussian function:

$$f(x,\mu,\sigma) = \frac{1}{Norm}\exp\left(-\frac{(x-\mu)^2}{2\sigma^2}\right) \quad ,$$

$$\frac{d\Sigma}{d\Omega}(Q) = \frac{volfrac}{V}\iint_0^\infty \int_0^{\frac{\pi}{2}} f(x_R,R,\sigma_R)f(x_L,L,\sigma_L)F^2(x_R,x_L,Q,\alpha)\sin(\alpha)\,d\alpha\ \,dx_R\,dx_L + B\ ,$$

whereas $Norm$ equals a normalization factor, μ the mean of the distribution and σ the standard deviation. The integrand was chosen to only contribute values of $\pm 3\sigma$ to decrease the load on the calculation. The radius and length were both fitted. To further constrain the scattering volume or volume fraction of particles, the last frame of the in-situ measurement was taken, and it was assumed that the number of particles per liter N stays the same over the whole experiment. N was calculated by dividing the volume fraction resulting from the fit of the final frame through the volume of one particle. The fit algorithm worked backwards from the final frame to the first one calculating the volume fraction corresponding to the fitted geometry of a single particle and the particle number per liter N. At around 35 minutes only background signal can be seen, resulting in unphysical values for radius and length, which is why those parameters are omitted in **Figure 9c** in the manuscript.

The SANS data was fitted by applying a well-defined model previously published by our group (Schmutzler *et al.*, 2018). The shape of the micelles can be described by using a core-shell ellipsoid model for the form factor of the scattered intensity:

$$F(Q,\alpha) = F_{core}\left(Q,R_{minor},R_{major},\Delta\rho_{core},\alpha\right) + F_{shell}\left(Q,R_{minor}+t,R_{major}+t,\Delta\rho_{shell},\alpha\right) \quad ,$$

$$F(Q,R_1,R_2,\Delta\rho,\alpha) = \frac{3\Delta\rho V\left(\sin\left(Qr(R_1,R_2,\alpha)\right)-\cos\left(Qr(R_1,R_2,\alpha)\right)\right)}{\left(Qr(R_1,R_2,\alpha)\right)^3} \quad ,$$

$$r(R_1,R_2,\alpha) = \sqrt{}\left(R_1^2\sin^2(\alpha) + R_2^2\cos^2(\alpha)\right) \quad ,$$

whereas R_{minor} is the minor half axis, R_{major} the major half axis and t the thickness of the shell of the core-shell ellipsoid. Furthermore, $\Delta\rho_{shell}$ is corresponding to the scattering contrast between dispersion medium and shell, $\Delta\rho_{core}$ is corresponding to the scattering contrast between core and shell, V is the volume of either the shell or core of the nanostructure and α is the angle between minor half axis and scattering vector $\vec{Q}$. To model the correlation peak between micelles a Hayter-Penfold structure factor was applied (Hayter & Penfold, 1981). Additionally, the shell thickness t of the form factor was

distributed by a gaussian function, which resulted in the following relation for the scattering cross section for randomly oriented particles and the corresponding structure factor $S(Q)$:

$$\frac{d\Sigma}{d\Omega}(Q) = \frac{1}{V}\, S(Q) \int_0^{\infty} \int_0^{\frac{\pi}{2}} f(x_t, t, \sigma_t) F^2(t, Q, \alpha) \sin(\alpha)\, d\alpha\ dx_t + B\ .$$

Here V equals the volume of the core shell ellipsoid $V = \frac{4}{3}\pi(R_{major} + t)(R_{minor} + t)^2$ and B the contribution of a constant background signal. The volume fraction, major half axis, charge of the micelles and salt concentration in the solution were fitted. As before the sequential fitting process started from the final frame to the first one. Predefined models from the package as models implemented in the program SasView (Doucet *et al.*, 2019) were used in combination with a custom-made python script to sequentially fit the in-situ SAXS and SANS data.

1.5. Transmission Electron Microscopy (TEM):

Microscopy images of the Au NPs synthesized in the experimental setup were carried out with a Philips CM30 transmission electron microscope. It was maintained at an acceleration voltage of 300 kV and utilized a LaB_6 cathode at a current of 24 mA. The samples were washed once more with H_2O, to reduce the amount of CTAB in the solution and drop-casted onto a copper grid covered with a thin carbon film (Plano).

2. Performance of the SAXS instrument

Following a solvent (H_2O) subtraction, the SAXS profiles are modeled using SASfit (Bressler *et al.*, 2015) with only a sphere form factor with a log-norm distribution function. No structure factor is applied to the fitting. According to the fitting, SiO_2 NPs is bimodal distributed with a diameter of 26±2 nm and small fraction of aggregates of about 70 nm in size.

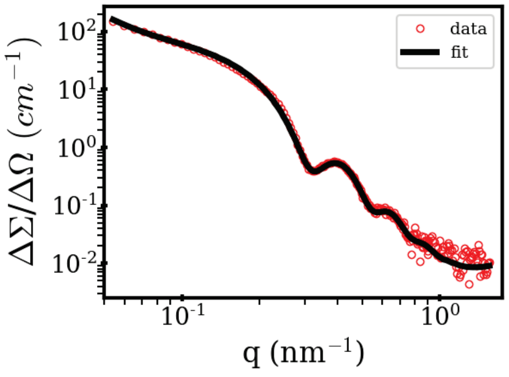

Figure 1S. 1D SAXS profiles (open circle) and the data fit (solid lines) of the Ludox TM50 (Si NPs) sample.

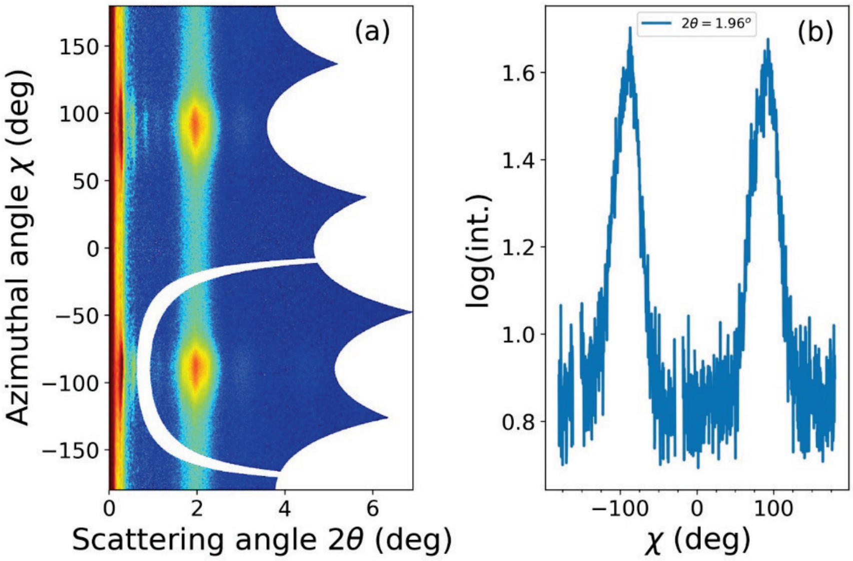

Figure 2S a) Azimuthal 2D regrouping, b) radial plot (at 2θ = 1.96°) of platelet-like shaped tripalmitin nanocrystals (TP NCs) collected at a SDD of 523 mm.

3. SAXS at two different sample angles

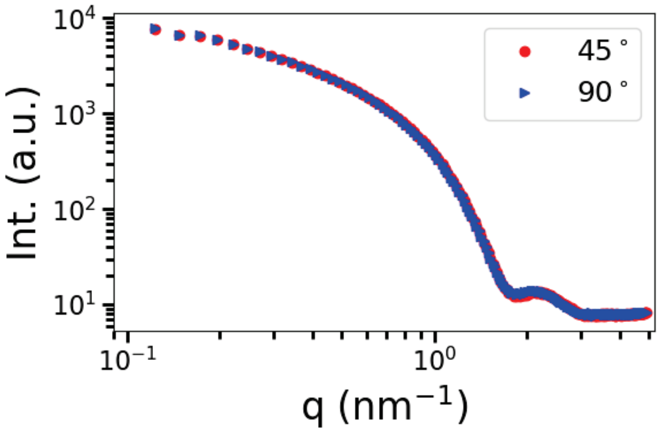

Figure 3S. 1D SAXS profiles of a CdSe nanoparticle sample. Correction of scattering profile, by multiplying each data point of the sample measured at 45° angle by 1.414, is perfectly coincidence with that collected at 90° angle.

4. Effect of lead shielding on the background intensity

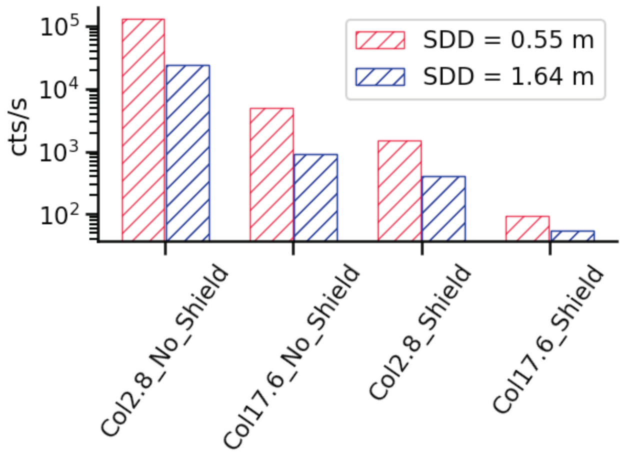

Figure 4S. The effect of lead shielding on the background intensity collected using a single-energy threshold (suppress all X-ray energy below 4 keV) detector while neutron shutter is opened for two different neutron collimations (short; collimation = 2.8 m and long; collimation = 17.6 m). All background measurements on the X-ray detector are repeated for both short and long sample-to-detector (SDD) distance. The intensity data are shown on a logarithmic scale.

5. Empty cell and water SAXS profiles:

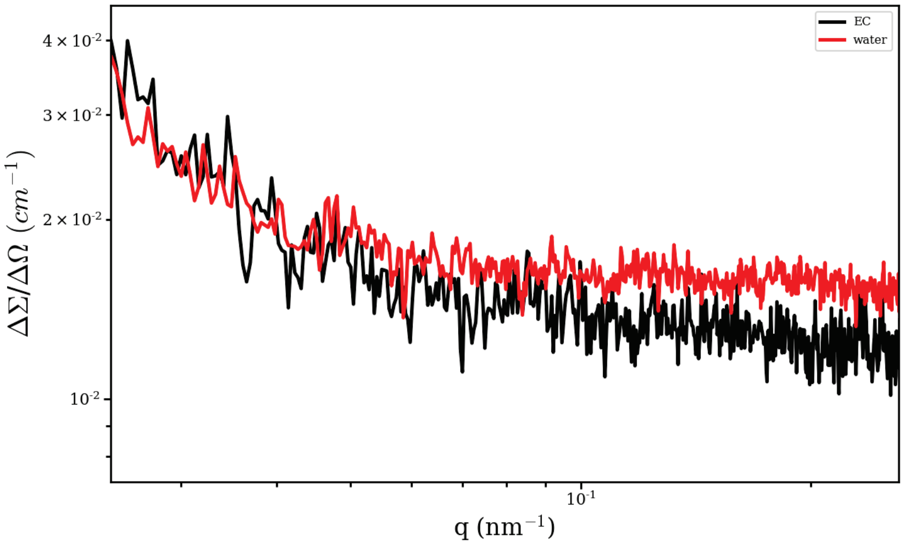

Figure 5S. Azimuthally averaged SAXS data of empty cell EC (black), a 1-mm thick water (red) sandwiched between two mica windows (cell).

6. Schematic drawing of gold nanorods formation:

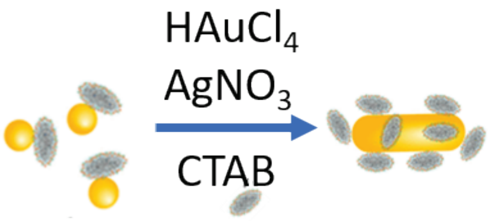

Figure 6S. Schematic drawing showing the reduction step of $HAuCl_4$ using Hydroquinone ($C_6H_6(OH)_2$) in the presence of $AgNO_3$, CTAB, and the seed particles leading to the formation of single crystalline Au NPs. It is adapted from the work of *Schmutzler, T. et al. (2019)*.

References:

Bressler, I., Kohlbrecher, J. & Thunemann, A. F. (2015). *J Appl Crystallogr* **48**, 1587-1598.

Doucet, M., King, S., Butler, P., Kienzle, P., Parker, P., Krzywon, J., Jackson, A., Richter, T., Gonzales, M., Nielsen, T., Ferraz, L., R., Markvardsen, A., Heenan, R., Bakker, J. & Alina, G. (2019). *SasView version 5.0.0,* https://www.sasview.org.

Hayter, J. B. & Penfold, J. (1981). *Mol Phys* **42**, 109-118.

Schmutzler, T., Schindler, T., Goetz, K., Appavou, M. S., Lindner, P., Prevost, S. & Unruh, T. (2018). *J Phys-Condens Mat* **30**.

Schmutzler, T., Schindler, T., Zech, T., Lages, S., Thoma, M., Appavou, M.-S., Peukert, W., Spiecker, E. & Unruh, T. (2019). *ACS Applied Nano Materials* **2**, 3206-3219.

Vigderman, L. & Zubarev, E. R. (2013). *Chem Mater* **25**, 1450-1457.

Zhang, F., Ilavsky, J., Long, G. G., Quintana, J. P. G., Allen, A. J. & Jemian, P. R. (2010). *Metall Mater Trans A* **41a**, 1151-1158.

Acknowledgements

This work benefited from the use of the SasView application, originally developed under NSF award DMR-0520547. SasView contains code developed with funding from the European Union's Horizon 2020 research and innovation programme under the SINE2020 project, grant agreement No 654000.
